# Work-related participation impairments and support needs of patients in a Swiss psychiatric university hospital

**DOI:** 10.3389/fpsyt.2023.1232148

**Published:** 2024-01-04

**Authors:** Niki Hug, Lukas Imfeld, Benjamin Holinger, Dorothea Jäckel, Christian G. Huber, André Nienaber

**Affiliations:** ^1^Department of Education, Research and Practice Development, Universitäre Psychiatrische Kliniken Basel, Basel, Switzerland; ^2^Vivantes Klinikum am Urban, Charité Klinik für Psychiatrie, Berlin, Germany; ^3^Zentralinstitut für Seelische Gesundheit (ZI) Mannheim, Mannheim, Germany

**Keywords:** work-related participation, return to work, inpatient psychiatric treatment, psychiatric disorder, job coaching

## Abstract

**Objective:**

To assess work-related participation impairments and support needs of adult patients in inpatient and day-care treatment at a Swiss psychiatric university hospital.

**Methodology:**

Cross-sectional survey on a department-dependent cut-off date in May and June 2022 using a standardized structured interview.

**Results:**

Data were available for 93 patients (response rate 59%), of which 51% (*n* = 47) stated that they had a job or training place. Patients in first hospitalization and with a job or training place were approached significantly more often. Regardless of age and first hospitalization, 76% of the patients expressed a need for support, of which 92% expressed interest in job coaching. A total of 54% of the patients stated that they had received support from the treatment team.

**Conclusion:**

From the patients’ point of view, work and education were not addressed by the treatment team across the board and independently of patient characteristics. The need for support was insufficiently met. There is a considerable interest for support programs through job coaching, and this offers opportunities to promote the inclusion of patients in the regular labor market.

## Introduction

The importance of work goes beyond mere subsistence and remuneration. Work can create identity, help people find meaning and value, enable self-efficacy and ultimately define social status (1, 2). Work and mental health are closely linked (3). Work serves as an important resource for maintenance and recovery of mental health (2). Conversely, unemployment has been shown to lead to clustered health care use due to increased psychological distress and physical discomfort, and is ultimately associated with higher mortality rates (4–6). Being out of work can have far-reaching consequences on activities and participation for those affected (7). For example, lower life satisfaction, stigmatization, loss of self-esteem and social contacts. People without work are 1.7 to 3.5 times more likely to suffer from mental health problems (3). Furthermore, unemployment can also have indirect effects on those affected, for example through a lower income or the associated loss of opportunities for participation and fixed daily and time structures (8, 9).

Education is considered an important protective factor against unemployment and for maintaining mental and physical health. The level of education affects the occupational status and thus also influences the working conditions and income, the individual health attitude and the health behavior of a person (3). Education enables a more competent handling of the structures of the health system and health-related information (10). It has been shown that people who have no education beyond compulsory education are more frequently affected by mental health problems (3). In addition, migration has been shown to be a possible cause of increased psychosocial stress due to lower socioeconomic status and poorer living and working conditions (11). Of the first-generation migrant population in Switzerland (around 30%), 20–29% report psychological complaints, depending on the age group (3).

International figures on employment rates for people with severe mental illness vary widely by country and region and may also be influenced by factors such as societal attitudes, policies and support systems in place. For schizophrenia in Europe, it is assumed that only 10–20% have a job (12). In the United States in particular, the figures are unclear. Studies in Germany showed that “effective interventions to promote work-related participation” are not used enough in inpatient psychiatric treatment (13). This is despite the fact that people with mental illness have an interest in working in the primary labor market (13, 14). Accordingly, only two thirds of people succeed in re-entering the workplace after leaving hospital without help (15). In this context, it is necessary to optimize the interface between the social insurance system and health care by specifically promoting reintegration (7, 16), as is also addressed in the report “The Future of Psychiatry in Switzerland” (17).

Current figures from the Swiss Disability Insurance (IV) indicate a trend reversal in the total number of insured persons. After the total number of all IV pensioners in Switzerland declined from 2005 to 2019, a steady increase has been recorded in the last 3 years. This is in particular caused by new pensions due to mental illnesses. Almost every second new pension is now granted due to a mental illness. In 2021, around 50% of the causes of disability for a pension were due to mental illnesses. This makes it the most frequent cause, ahead of other illnesses (31%), birth defects (13%) and accidents (6%) (18). This shows that current measures to improve the participation situation of people with mental illnesses are currently not effective enough or are taken too late. Early support on the topic of work is central to preventing long-term exclusion from the labor market (19, 20).

In a survey of 176 patients in inpatient treatment at a Berlin clinic, Jäckel et al. (13) showed that 63% of the patients surveyed had a need for support. The interest in participating in job coaching was also correspondingly high. Furthermore, the results of the survey show that 49% of the patients had been approached about the topic of work. Overall, however, only 20% received concrete offers of help. According to the results, people who were ill for the first time and young adults in particular indicated a strong need for support (13). On admission to hospital, 34% (13) of psychiatric hospitalized persons had a job or training place. In view of the importance of the topic, especially in the context of mental illness, the authors point to an urgent need for action.

Work is an important issue in the treatment of people with mental illness (1, 21, 22). It should be considered and dealt with at an early stage in the context of psychiatric treatment in order to be able to agree on and initiate measures to support work and professional employment if necessary (21). It seems to be important to identify the needs of the patients. Studies on work-related participation impairments and a work-related need for support in day-care and inpatient psychiatric treatment do not yet exist for Switzerland.

The aim of the present study was to assess work-related impairments in participation and the need for support among adult patients in day-care and inpatient treatment at a Swiss psychiatric university hospital. In addition, it was to be determined whether there are group differences within the sample with regard to the expressed need for support and the support received in terms of socio-demographic characteristics (age, educational level, migration background), diagnosis, first-time hospitalized patients and patients with or without a job/training place.

## Materials and methods

The UPK Basel has a mandate to provide care for the canton of Basel-Stadt with a catchment area of about 195,000 inhabitants. They provide treatment services for adult (UPKE) and child and adolescent psychiatry (UPKKJ), and have a forensic clinic and a private clinic. This ensures treatment for the entire spectrum of psychiatric illnesses. For the inpatient care of patients, around 300 inpatient treatment places are available at the UPK Basel in the four specialized clinics.

The anonymous patient survey was classified by the Ethics Committee Northwestern and Central Switzerland (EKNZ) as quality assurance not subject to approval. All patients aged 18–65 years who had been in inpatient treatment for at least 5 days in the survey period May/June 2022 and who agreed to participate in the study were taken into account. During the survey period, a cut-off date was formulated for each department, on which the survey was conducted in each case. A total of 15 departments of the UPKE and the private clinic were included.

The only exclusion criterion was florid psychotic symptoms. The fulfillment of the inclusion and exclusion criteria was assessed in each case by a responsible specialist of the corresponding department (as a rule, the respective head of the nursing department). The computer-assisted key date survey was carried out by various specialists from nursing and social services. In order to ensure a standardized implementation of the survey, the interviewers were instructed in a session and an online tutorial with an exemplary survey was made available.

The survey was conducted as an interview using a version of the standardized survey instrument by Jäckel et al. (13) adapted for Switzerland. The adapted survey instrument can be found in Supplementary Appendix 1. The central questions from it are listed below.

- “Are you currently in regular employment or training?”

– if “no”: Is employment/training an issue for you in principle?

- “Do you need help with re-entering employment (work/training)/finding a suitable job/training place, or with vocational (re)orientation?”
- “Do you have any concerns about taking up (re-entering) employment/training?”
- “Which areas do you worry about taking up (re-entering) work/training?”
- “Have you been approached or encouraged to approach the topic of work/training here in the clinic (UPK)?”
- “Have you already received concrete support in the clinic (UPK) on the subject of work/training?”
- “Which path (direct entry into the 1st labor market with support or gradual entry in a protected environment for the time being) or do you prefer for (re-)entry?”
- “Would you be interested in taking part in a job coaching/support programme?” [note: individualized job coaching according to supported employment and its fidelity scale (23)]

It was possible to interrupt or discontinue the survey at any time. In addition, the highest level of education, migration background and psychiatric utilization (first inpatient treatment) were collected. The information on psychiatric main and secondary diagnoses, age and gender was taken from the hospital information system.

The analysis plan was based on the comparative study from Germany (13) and was designed as follows: The central questions on the “work-related participation situation,” the “need for support,” “having been approached about the topic during the stay,” “having received concrete help during the stay” and the question on “concerns about resuming work” were coded into “presence” and “absence.” Subsequently, it was analyzed for each characteristic whether the distribution of answers differed between the different groups. The groups studied were (1) patients with work and patients without work, (2) patients up to 35 years of age and patients over 35 years of age, (3) first hospitalized and re-hospitalized and (4) F-main diagnostic groups, (5) educational attainment and (6) migration background. The two age groups were defined on the basis of the average age of the sample (*M* = 36.0) and correspond to those in the analyses of the comparative study (13).

Within the framework of descriptive statistics, absolute (n) and percentage figures (%), the arithmetic mean (M) and standard deviation (SD) were calculated. To calculate the group comparisons for categorical variables, *χ*^2^-tests were applied. For continuous variables, t-tests and Pearson correlation were applied. All statistical analyses were performed using SPSS Statistics version 27. The significance level was set at *p* < 0.05. In view of the limited number of cases and the pilot study character, a correction for multiple testing was waived.

## Results

A total of 158 out of 192 patients met the inclusion criteria, of which 58.9% (*n* = 93) agreed to participate in the survey and could be interviewed. The description of the sample with socio-demographic and work-related characteristics is shown in Table 1. The characteristics of the sample were matched with the population of partial and full inpatients in the UPK. The average age (M) was significantly lower in the sample (*M* = 36.0; *M* = 40.8), and patients with an F6 (sample: 26%; population: 8%) or an F3 diagnosis (sample: 40%; population: 27%) were overrepresented in percentage terms, while F1s were underrepresented (sample: 16%; population: 39%).

**Table 1:** Sample description.

Sample description (*n* = 93)		Population description (Patients in the UPK may/june 2022)	
Age (M ± SD)	36.0 ± 12.7	40.8 ± 12.5	
Gender: male	51 (55%)	54%	
In work/training/study ≥15 h/week	47 (51%)	(data not available)	
Diagnose:			
F1.X	15 (16%)	F1.X	39%
F2.X	10 (11%)	F2.X	13%
F3.X	37 (40%)	F3.X	27%
F4.X	4 (4%)	F4.X	8%
F5.X	2 (2%)	F5.X	1%
F6.X	24 (26%)	F6.X	8%
F8.X	1 (1%)	F8.X	0%
		Others	4%
Comorbidity	62 (67%)	(data not available)	
In inpatient treatment for the first time	54 (58%)	(data not available)	
Not in work/training/study and without interest in (re-)starting work/training/study	9 (10%)	(data not available)	
Educational attainment:			
Compulsory school	23 (25%)	Compulsory school	28%
Secondary level II	38 (42%)	Secondary level II	42%
Tertiary level	30 (33%)	Tertiary level	21%
		No completed school education	9%
Migration:		(data not available)	
No migration background	54 (58%)		
Migration background 1st generation	18 (19%)		
Migration background 2nd generation	21 (23%)		

### Work-related participation situation

At the time of the key date survey, 49.5% (*n* = 46) were without work or without regular training or studies (hereafter: work). This status was significantly related to the highest level of education (compulsory school/secondary level II/tertiary level) (*χ*^2^ [2.91] = 14.1; *p* < 0.01). Among the patients with compulsory school as their highest qualification, 83% (*n* = 19) were without a job. In Switzerland, compulsory school lasts 11 years and consists primary school (incl. kindergarten) and secondary school level I.

Of those without work, 80% (*n* = 36) stated that work was a relevant issue for them. Regarding age, for 8% (*n* = 2) of those under 36, work was basically not an issue; for those over 35, it was not an issue for 35% (*n* = 7) (*χ*^2^ [1.45] = 5.06; *p* < 0.05).

At the time of the survey, 10% (*n* = 9) of the patients were not in work and at the same time stated that work was basically not an issue for them. The reasons given for this were, on the one hand, an already existing financial security, the feeling of not being able to or having no chances on the labor market, or working in a sheltered workshop without interest in a job in the primary labor market. In the following evaluations of the need for support, patients without work and without interest in work were not taken into account to ensure comparability with the study by Jäckel et al. (13).

Whether the patients were approached about the topic of work correlated significantly with the length of stay in number of days (*r* = 0.26, *p* = 0.14, *n* = 93). According to Cohen (24), this is a weak to medium effect.

### Need for support with work-related participation and assistance from the treatment team

A total of 76% (*n* = 63) of patients expressed a need for support to (re)start work. Patients who were not currently in work indicated significantly more need for support (89%; *n* = 32; *χ*^2^ [1.83] = 5.86; *p* < 0.05) than those with work (66%; *n* = 31). There were no significant differences with regard to age or whether patients were hospitalized the first time or not.

A share of 31% (*n* = 29) of the respondents preferred a direct entry into the first labor market (first place) to a gradual (re-)entry with training and preparation (40%; *n* = 37). 29% (*n* = 27) did not choose either option. Further, 79% (*n* = 73) of the interviewed patients were interested in a specific support program (“job coaching”).

The interest in job coaching did not depend significantly on age or whether the patients were in work.

Overall, 77% (*n* = 72) were approached by a professional from the treatment team about the topic of work. Patients who were working (57%; n = 41) were approached significantly more often (*χ*^2^ [1.93] = 5.24; *p* < 0.05) than patients without work/training. Also, those who were hospitalized for the first time were approached significantly more often (64%; *n* = 46; *χ*^2^ [1.93] = 4.44; *p* < 0.05) than patients who were hospitalized more than once. In total, 71% were addressed by a social worker. A thematisation by other professional groups varied strongly, depending on the department (0–100%).

54% (*n* = 39) stated that they had received concrete help in (re-)starting work. Those over 35 years of age were significantly more likely to report having received help (71%; *n* = 20) than those under 36 years of age (43%; *n* = 19; *χ*^2^ [1.72] = 5.50; *p* < 0.05). 90% of patients reported that they had received help from a social worker. Nursing staff (28%), psychologists (23%) and doctors (28%) were also mentioned. The help included, in particular, advice on direct (49%) or gradual/delayed (50%) (re-)entry into a job on the primary labor market as well as contacting/conversing with employers (31%).

In total, 78% (*n* = 36) of the patients with work stated that they had moderate to strong concerns about returning to work. For those without work (unemployed or retired), this was 64% (*n* = 21). Regarding the number of worries, patients of age over 35 years reported significantly more worries (*M* = 2.79; SD = 1.31; *n* = 28) compared to those of age under 36 years (*M* = 2.02; SD = 1.09; *n* = 47).

### Diagnosis-related thematisation of work-related participation, support needs and assistance by the treatment team

**Figure 1:**
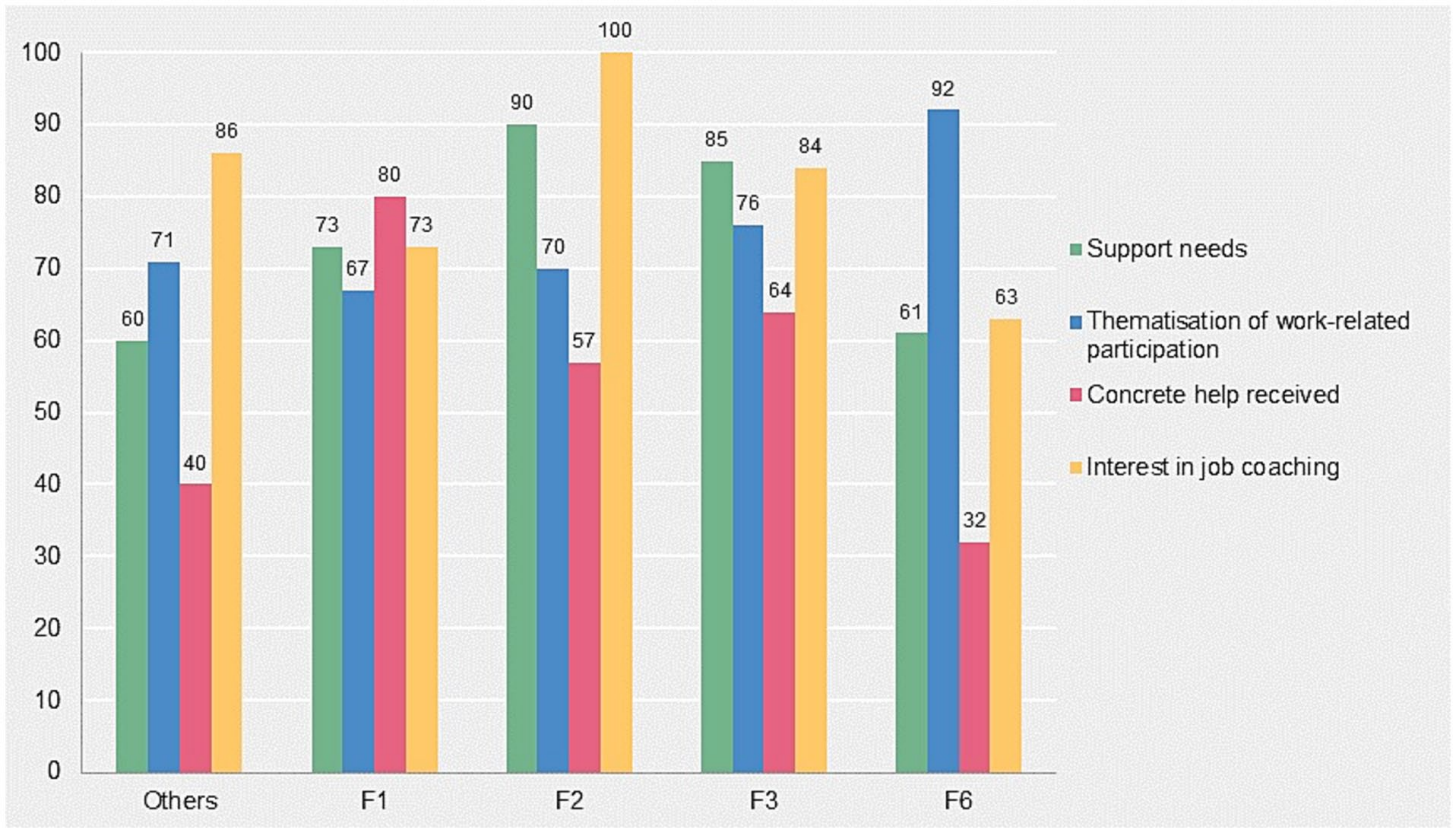
Support needs, thematisation of work-related participation, coverage of concrete help and interest in job coaching grouped by ICD-10 diagnoses (in%).

With regard to the main diagnostic groups, differences were found with regard to work-related characteristics (Figure 1). Patients with an F6 diagnosis stated significantly more often that they had been approached about the topic of work (92%; *n* = 22) than patients with diagnostic groups F1, F2, F3, and other psychiatric disorders (72.5; *n* = 50; *χ*^2^ [1.93] = 3.76; *p* < 0.05). At the same time, significantly less need for support was reported by the group with an F6 diagnosis (61%; *n* = 14) compared to the other diagnostic groups (82% *n* = 49; *χ*^2^ [1.83] = 3.9; *p* < 0.05), as was help received by the group with an F6 diagnosis (32%, *n* = 7) vs. the other diagnostic groups (64%; *n* = 32; *χ*^2^ [1.72] = 6.36; *p* < 0.05). Interest in participating in job coaching was also significantly lower among those with an F-6 diagnosis (63%; *n* = 15) vs. the other diagnostic groups (84%; *n* = 58; *χ*^2^ [1.93] = 4.9; *p* < 0.05).

For the diagnosis-related results, a high number of comorbidities should be mentioned here. 67% (*n* = 62) of the patients had a psychiatric comorbidity due to at least two diagnoses from different F-main diagnostic groups. Patients with a diagnosis from section F6 (92%) and section F2 (70%) showed the highest comorbidity rates.

### Thematisation of work-related participation, support needs and assistance by the treatment team in the different diagnostic groups

The group comparison of patients with support needs versus without support needs (Table 2) showed that the concrete help received and the interest in job coaching were significantly higher among patients with support needs. Patients without support needs, however, stated more frequently that they had been approached about the topic of work. The presence of at least moderate concerns and the number of concerns and worries, on the other hand, were not significantly higher among those who expressed a need for support.

**Table 2:** Group comparison for patients with support needs and patients without support needs.

Group comparison between patients with support needs and patients without support needs (only patients with work or assessment of work as relevant *n* = 83)			
	Patients with support needs	Patients without support needs	Significance
Work addressed	49 (78%)	19 (95%)	0.08
Moderate to very strong concerns about (re-)starting work	44 (72%)	13 (65%)	0.54
Number of concerns about (re-)starting work	*M* = 2.34; SD = 1.20	*M* = 2.19; SD = 1.38	0.67
Concrete help received	31 (63%)	6 (32%)	0.02
Interest in job coaching	58 (92%)	11 (55%)	0.01

There were no significant differences between the group of patients with a migration background and those without a migration background for any of the work- and support-related variables.

## Discussion

With regard to a slight bias in the selection of the sample in terms of age and diagnoses, the present data were able to provide an insight into the patient perspective on the assessment of work-related participation limitations and the associated support needs in a Swiss psychiatric university hospital.

In comparison with the survey by Jäckel et al. (13), the present results of the key date survey also showed a very high level of interest in the topic of work on the part of the patients surveyed, while at the same time there was an unmet need for support. This is despite the fact that the topic of work was addressed more frequently in the treatment of patients at the UPK (77%) and with 42% twice as many participants stated that they had received work-related assistance compared to the survey in the psychiatric hospital in Germany. The socio-demographic data on age and gender were comparable at the two sites. The proportion of patients with jobs was also significantly higher at 51% compared to Jäckel’s (13) proportion of 34%. This discrepancy could indicate differences in the Swiss and German care systems, as in the latter psychiatry and psychosomatics are organized in different care systems. Overall, a key difference between the two care systems is that the welfare system in Germany is fundamentally more fragmented than in Switzerland. While in Switzerland, support for reintegration is provided exclusively by the disability insurance (IV), in Germany there are various cost bearers (SGBs), which can potentially lead to more gaps in the care system. However, the study did not focus on differences in the care systems. Comparisons between Germany and Switzerland with regard to different interventions and concepts can be found in various places in the literature (25, 26). Nevertheless, this could also account for the differences in the distribution of diagnoses, the significantly smaller group with an F2 disorder in our sample (8% versus 24%) with strong similarity in the two hospitals for the other diagnostic groups. Mental comorbidities were significantly higher in our survey (67% versus 48%). The high proportion of psychiatric comorbidity is remarkable, as somatic comorbidities were still excluded. The issue of work was considered relevant for almost all young adults. However, an increased need for support especially among young adults and first-time sufferers could not be identified in our study.

With regard to potential differences in the population of the two healthcare service areas, the average unemployment rate in Germany at 5.3% (27) in 2022 was only slightly higher than in Switzerland at 4.3% (28) in the same year and does not explain the significantly higher number of unemployed people with a mental illness, mentioned by the study in Germany (13). However, the different poverty quotient in the two coverage areas of the two studies differ significantly, which limits comparability. For the urban coverage area of the survey in Germany, Berlin in the district of Friedrichshain-Kreuzberg, a poverty rate of 19.7% (29) is given. In densely populated areas of German-speaking Switzerland, on the other hand, the poverty rate is assumed to be 10.9% (30). As mentioned at the beginning, mental illness, poverty and the ability to work are closely linked. It could also be argued that the label “university” of the psychiatric clinic in our study has a positive influence on expectations regarding support for work integration. Even though the clinic in Germany, Berlin, the *Vivantes Klinikum am Urban*, is also an academic teaching hospital of the *Charité - Universitätsmedizin Berlin*, the label is less obvious and therefore the influence could be smaller. However, this cannot be investigated with our data, but should be considered for future studies.

The interest in participating in job coaching was also very high in our survey. This is an important prerequisite for implementing evidence-based approaches to (re)integration, such as “Individual Placement and Support” (IPS), i.e., direct entry into the first labor market with individual and long-term support (31). Contrary to the study in Germany (13), some of the patients surveyed in our sample were being treated in private wards (n = 10). It is known that people with private or semi-private insurance in Switzerland have a higher salary, higher education and are older than people with general insurance (32). As a result, it can be hypothesized that their work situation and requirements differ systematically. However, it must be borne in mind that (semi-)privately insured persons in Switzerland are not such an exclusive group at 29% (32). Our data shows that although the group with privately insured patients are on average more likely to have a job (60% versus 51%), the need for support is similar to that of the public/general departments (80% versus 76%).

What was striking and deviating from the results of the Berlin study (13) was the diagnosis-specific evaluation in our survey, especially for those with a main diagnosis from the field of personality and behavioral disorders. For these patients, the topic of work was addressed significantly more often than for the other diagnostic groups. At the same time, these patients expressed less need for help than the other diagnostic groups and only about half of them stated that they were offered support. In addition to a diagnosis-specific interpretation, the reasons for this could also lie in department-typical processes and structures. For the comparison with the results of the other diagnoses, it must also be considered that the F1 diagnoses were overrepresented (see Table 1). In any case, it became clear that despite a routine thematisation (91%) of the work-related participation situation, the need for support may be insufficiently covered. In this context, it was also noticeable that especially patients with work and first-time hospitalized persons were addressed on the topic of work, but patients without work and multiple hospitalized persons also expressed a high need for support. The current practice thus bears the risk of not providing sufficient care for patients who have already fallen out of work or chronically ill persons, since the topic of work is not sufficiently addressed in this group. However, it could be that for patients in a particularly precarious situation, topics such as housing and crisis management may have priority and the topic of work is therefore only taken up in a more stable situation. The results show that the discussion of work increases with the length of stay.

It was also found that patients who were in employment or had a training place as well as older patients expressed more concerns about (re)starting their training or job. Possible reasons for this were uncertainty as to whether these patients would be able to resume their activities after the inpatient stay or whether retirement would become necessary. Whereas for those without a job or training place, in some cases a pension had already been taken and this possibly led to fewer worries due to the clarified situation (excluded from the general labor market). Addressing concerns about the loss of a job or training place at an early stage and taking appropriate measures can not only lead to an increase in patient satisfaction, but job retention is also associated with less effort and better prospects of success for long-term employment in the first labor market than is the case with reintegration after job loss (19).

The finding that people with higher education are more likely to be employed in the labor market was confirmed. In addition, education and migration did not show any systematic differences with regard to the thematisation of work or the need for support.

The present study was able to show that work and training are highly relevant for people with a mental disorder and that there is a great interest in (re)entering employment in the primary labor market. Against the background of an increase in mentally related retirements in Switzerland in recent years (17), the urgency of routinely addressing work and training in the context of psychiatric treatment and providing assistance to discuss and initiate appropriate measures to support work and professional employment as early as possible (18) becomes all the more clear. Evidence-based programs of Supported Employment such as the most clearly described and most frequently evaluated is the IPS model. It offers promising evidence to reduce barriers to access to the world of work for patients with mental illness and to promote inclusion and recovery (7, 33). The core principles of IPS include rapid placement in the primary labor market, systematic development in the workplace, consideration of individual preferences, integration of mental health and employment services, and support from specialized services (34). Studies suggest that IPS can double the chances of permanent employment in the primary labor market (30, 33, 34). However, due to the difficulty of finding a suitable job in the first place, IPS approaches combined with skills training around job search and retention, such as social skills training (35, 36) or cognitive remediation training (37), are proving most effective. To this end, adjustments to the Swiss social security system would also be beneficial by allowing part-time work of less than 50% in the first labor market with a simultaneous IV pension (38). Although Switzerland has a very well-developed vocational training system, particularly with regard to the combination of training and work, the labor market results for low-skilled workers are poor (8). This also affects people who often suffer from a mental disorder. The relatively high full and extraordinary IV pensions are not beneficial to entering the labor market due to lack of incentives. There may be people for whom the primary labor market is not the right place since they are dependent on protection, despite integration measures (39). Also, the barriers to accessing a sheltered job are lower in Switzerland than in Germany (meeting the criteria for an IV pension is sufficient in Switzerland). However, sheltered jobs are often associated with lower income, less social integration and low professional prestige (40). In this regard, there is certainly a need to upgrade and adapt sheltered jobs too.

Ultimately, against the backdrop of the sharp rise in healthcare costs in Switzerland and the significant undersupply of mentally ill people in Switzerland (41) it is becoming increasingly clear how important it is to implement the most effective concepts in practice.

## Limitations and future research needs

The survey collected the subjective patient perspective. A general discussion of the results with the treatment team was implemented, but a systematic study with the comparison of the patients’ and the treatment team’s perspectives would be exciting to uncover discrepancies, especially in the perception of support, and at best to show reasons for a lack of discussion of work. It remains open, for example, how patients define the help they receive from the treatment team. Consequently, it is possible that the patients did not perceive help as such from the treatment team’s point of view. For future surveys, we recommend a prior definition of assistance for all survey participants. Furthermore, the sample is slightly biased with regard to age and diagnosis, which can be explained by the voluntary nature of the survey. A routine survey in the context of treatment could increase the representativeness of the population. It also remains open which influencing factors of the different care systems (Basel, Switzerland and Berlin, Germany) have caused the partially discrepant results in the comparison.

### Consequences for clinical practice

The issue of work should be addressed in treatment regardless of age, diagnosis, frequency of hospitalization and place of work/training.

Measures are needed to minimize the expressed discrepancy between desired support and effective assistance through appropriate interventions.

Patients express great interest in evidence-based support for work-related participation such as Individual Placement and Support (IPS), i.e., job coaching with direct entry into the primary labor market. In practice, these programs should be increasingly implemented.

## Data availability statement

The raw data supporting the conclusions of this article will be made available by the authors, without undue reservation.

## Ethics statement

The studies involving humans were approved by Ethics Committee Northwestern and Central Switzerland (EKNZ). The studies were conducted in accordance with the local legislation and institutional requirements. Written informed consent for participation was not required from the participants or the participants’ legal guardians/next of kin in accordance with the national legislation and institutional requirements. Written informed consent was obtained from the individual(s) for the publication of any potentially identifiable images or data included in this article.

## Author contributions

NH, LI, BH, and AN: substantial contributions to the conception and design of the work, and the acquisition, analysis, and interpretation of data for the work. DJ, and CH: revising the article critically for important intellectual content. All authors contributed to the article and approved the submitted version.
