# Supplementary material for: Work-related participation impairments and support needs of patients in a Swiss psychiatric university hospital
Source: Front Psychiatry. 2024 Jan 4;14:1232148. doi: 10.3389/fpsyt.2023.1232148 (PMC10794531; doi:10.3389/fpsyt.2023.1232148)
Supplement: Supplementary file 1 [file Data_Sheet_1.docx]

**Befragung zur beruflichen Situation und Bedürfnissen**

Patienten FID: _____________ Datum: _____________ Abteilung: _________________

Arbeit ist für fast alle Menschen ein wichtiges Thema, da Arbeit von vielen Menschen als sinnstiftend erlebt wird. Wir möchten aus diesem Grund auch von Ihnen erfahren, welchen Stellenwert Arbeit in Ihrem Leben hat. Hierzu möchten wir mit Ihnen ein kurzes Gespräch führen und bitten Sie, uns einige Fragen zu beantworten.

1. **Sind Sie erstmalig in psych. Behandlung in den UPK?**

⃝ ja ⃝ nein

1. **Sind Sie derzeit in einem regulären Arbeitsverhältnis (1. Arbeitsmarkt) oder in einer Ausbildung (Studium / Berufslehre)?**

*Keine Werkstätten für Menschen mit Beeinträchtigung, Rehabilitationseinrichtungen, Beschäftigungsprogramme, obligatorischen Schule.*

⃝ ja (weiter zu Frage 3a, links)

⃝ nein (weiter zu Frage 3)

**3) Ist eine Beschäftigung/ Tätigkeit in einem regulären Arbeitsverhältnis / in einer Aus- oder Weiterbildung/ in einem Studium / Berufslehre für Sie grundsätzlich ein Thema?**

⃝ ja ⃝ nein

| **WENN Ja** | **WENN nein** | |
| --- | --- | --- |
| **3a) In welcher Branche?** *(keine Mehrfachnennung)*  ⃝ Bau ⃝ Detailhandel  ⃝ Sicherheit ⃝ Medien  ⃝ Tourismus ⃝ Marketing/Werbung  ⃝ Bildung/Unterricht ⃝ Gastgewerbe  ⃝ Banken/Versicherungen  ⃝ Maschinen/Elektro/Metallindustrie  ⃝ Chemie/Kunststoff/Papier  ⃝ Gesundheitswesen  ⃝ Gebäudetechnik  ⃝ Kunst/Design  ⃝ Andere ______________  ⃝ Weiss nicht  **3b) wünschen Sie sich Hilfe …**  **... beim Wiedereinstieg in eine Beschäftigung (Arbeit/Studium/Lehre) / Suche eines geeigneten Arbeitsplatzes/ Studium, Ausbildung bzw. … bei der beruflichen (Um)Orientierung/ Orientierung für ein Studium, eine Ausbildung?**  ⃝ ja ⃝nein | **3e) Warum ist es kein Thema für Sie?** *(erstmal offen antworten lassen)*  _____________________________________________  _____________________________________________  _____________________________________________ | |
| **3c)** **Wie stark sind Ihre Bedenken eine Arbeit/ Ausbildung/ ein Studium aufzunehmen (wiederaufzunehmen)?**  ⃝ sehr stark ⃝ stark  ⃝ mäßig ⃝ kaum vorhanden  ⃝ nicht vorhanden  **3d) Welche Bereiche machen Ihnen Sorgen eine Arbeit/ Ausbildung/ ein Studium aufzunehmen (wiederaufzunehmen)?** *(Zunächst frei antworten lassen! Mehrfachnennungen möglich)*  ⃝ Reaktionen der Kolleginnen und Kollegen.  ⃝ Arbeit nicht mehr zu schaffen.  ⃝ erneut krank zu werden.  ⃝ als wenig belastbar eingeschätzt zu werden.  ⃝ Arbeit zu verlieren.  ⃝ ___________________________________ | **3f) Weil ich…** *(Zutreffendes bitte ankreuzen! Mehrfachnennungen möglich)*  ⃝ finanziell abgesichert bin. (IV-Teil-Rente,  Sozialhilfe, durch andere Personen).  ⃝ mich nicht fähig dazu fühle. (zu beeinträchtigt).  ⃝ keine Chance in einem regulären Arbeitsverhältnis  habe.  ⃝ eine Teilrente beziehe und darüber hinaus  beschäftigt bin.  **⃝** in einer Werkstatt für Menschen mit  Behinderung/ Reha-Maßnahme/Tagesstätte arbeite.  ⃝ ___________________________________ | |
| **WENN Ja** | | **WENN nein** |
| **4a)** **Wurden Sie bereits auf das Thema Arbeit/ Ausbildung/ Studium hier in der Klinik (UPK) angesprochen/ ermuntert es anzugehen?** *(Dies kann sich auch auf einen vergangenen Klinikaufenthalt beziehen.)*  ⃝ ja ⃝ nein *(weiter zu Frage 6)*  **WENN JA:**  **4b) Von wem wurden Sie auf das Thema hier angesprochen/ ermuntert es anzugehen?** *(Mehrfachnennung möglich)*  ⃝ Pflegepersonal ⃝ Arzt/ Ärztin  ⃝ PsychologIn ⃝ SozialarbeiterIn  ⃝ IPS – Jobcoach ⃝ PeerberaterIn  ⃝ Integrationsfachperson (IV)  ⃝ andere TherapeutIn (Arbeit, Ergo, Physio, Musik, Bewegung, Kunst) | | **4a) Obwohl es kein Thema für Sie ist, möchten wir Sie trotzdem standardmäßig fragen ob Sie bereits auf das Thema Arbeit/ Ausbildung/ Studium hier in der Klinik (UPK) angesprochen worden sind?** *(Dies kann sich auch auf einen vergangenen Klinikaufenthalt beziehen.)*  ⃝ ja ⃝ nein *(weiter zu Frage 6)*  **WENN JA:**  **4b) Von wem wurden Sie auf das Thema hier angesprochen/ ermuntert es anzugehen?**  *(Mehrfachnennung möglich)*  ⃝ Pflegepersonal ⃝ Arzt/ Ärztin  ⃝ PsychologIn ⃝ SozialarbeiterIn  ⃝ IPS – Jobcoach  ⃝ Integrationsfachperson (IV)  ⃝ PeerberaterIn  ⃝ andere TherapeutIn (Arbeit, Ergo, Physio, Musik, Bewegung, Kunst) |
| **5a) Haben Sie hier in der Klinik (UPK) bereits zum Thema Arbeit/ Ausbildung/ Studium konkrete Unterstützung erhalten?**  *Dies kann sich auch auf einen vergangenen Klinik-aufenthalt beziehen.*  ⃝ ja ⃝nein  **WENN JA:**  **5b) Von wem haben Sie konkrete Unterstützung erhalten?** *(Mehrfachnennung möglich)*  ⃝ Pflegepersonal ⃝ Arzt/ Ärztin  ⃝ PsychologIn ⃝ SozialarbeiterIn  ⃝ IPS – Jobcoach  ⃝ Integrationsfachperson (IV)  ⃝ PeerberaterIn  ⃝ andere TherapeutIn (Arbeit, Ergo, Physio, Musik, Bewegung, Kunst) | | **5a) Haben Sie hier in der Klinik (UPK) bereits zum Thema Arbeit/ Ausbildung/ Studium konkrete Unterstützung erhalten?**  *Dies kann sich auch auf einen vergangenen Klinik-aufenthalt beziehen.*  ⃝ ja ⃝nein  **WENN JA:**  **5b) Von wem haben Sie konkrete Unterstützung erhalten?** *(Mehrfachnennung möglich)*  ⃝ Pflegepersonal ⃝ Arzt/ Ärztin  ⃝ PsychologIn ⃝ SozialarbeiterIn  ⃝ IPS – Jobcoach ⃝ PeerberaterIn  ⃝ Integrationsfachperson (IV)  ⃝ andere TherapeutIn (Arbeit, Ergo, Physio, Musik, Bewegung, Kunst) |
| **5c)** **Die Hilfe/ Unterstützung die ich hier zum Thema Arbeit/ Job / Ausbildung/ Studium erhielt, war:**  ⃝ Beratung zur Berufswahl/ Praktika/ Ausbildung/ Studium.  ⃝ Beratung zur Wiederaufnahme einer bestehenden Arbeit im 1. Arbeitsmarkt.  ⃝ Beratung zur beruflichen Neuorientierung.  ⃝ Hilfe beim Bewerbungsschreiben / Stellensuche (auch Praktika, Brückenangebote).  ⃝ Kontaktaufnahme / Gespräche zu ArbeitgeberInnen.  ⃝ Beratung zu einer (Arbeits-) Rehabilitationsmaßnahme.  ⃝ Beratung zu (Rente/-) Teilrente, Tagesstätte, “geschützter Arbeitsplatz“ (Werkstatt für Menschen mit Behinderung).  ⃝ ____________________________________ | | **5c)** **Die Hilfe/ Unterstützung die ich hier zum Thema Arbeit/ Job / Ausbildung/ Studium erhielt, war:**  ⃝ Beratung zur Berufswahl/ Praktika/ Ausbildung/ Studium.  ⃝ Beratung zur Wiederaufnahme einer bestehenden Arbeit im 1. Arbeitsmarkt.  ⃝ Beratung zur beruflichen Neuorientierung.  ⃝ Hilfe beim Bewerbungsschreiben / Stellensuche (auch Praktika, Brückenangebote).  ⃝ Kontaktaufnahme / Gespräche zu ArbeitgeberInnen.  ⃝ Beratung zu einer (Arbeits-) Rehabilitationsmaßnahme  ⃝ Beratung zu (Rente/-) Teilrente, Tagesstätte, “geschützter Arbeitsplatz“ (Werkstatt für Menschen mit Behinderung).  ⃝ ____________________________________ |

| **WENN Ja** | **WENN nein** |
| --- | --- |
| **6) Wenn sie an ihren beruflichen (Wieder-) Einstieg denken, welcher Weg würde Ihnen eher entsprechen? Bei welchem dieser zwei Möglichkeiten fühlen sie sich sicherer und haben den Eindruck, dass Ihnen der (Wieder-) Einstieg besser gelingen würde?**  ⃝ Direkter (Wieder-) Einstieg am 1. Arbeitsmarkt/Ausbildung/Studium mit persönlicher und langfristiger Unterstützung  ⃝ Stufenweiser/verzögerter (Wieder-) Einstieg in den 1. Arbeitsmarkt/Ausbildung/Studium in geschützter Umgebung mit Training und Vorbereitung?  ⃝ Kein, Andere: (Freitext) | **6) Wenn sie an ihren beruflichen (Wieder-) Einstieg denken, welcher Weg würde Ihnen eher entsprechen? Bei welchem dieser zwei Möglichkeiten fühlen sie sich sicherer und haben den Eindruck, dass Ihnen der (Wieder-) Einstieg besser gelingen würde?**  ⃝ Direkter (Wieder-) Einstieg am 1. Arbeitsmarkt/Ausbildung/Studium mit persönlicher und langfristiger Unterstützung  ⃝ Stufenweiser/verzögerter (Wieder-) Einstieg in den 1. Arbeitsmarkt/Ausbildung/Studium in geschützter Umgebung mit Training und Vorbereitung?  ⃝ Kein, Andere: (Freitext) |
| **7) Wären Sie interessiert, an einem solchen Unterstützungsprogram teilzunehmen?**  ⃝ ja ⃝ nein | **7) Wären Sie interessiert, an einem solchen Unterstützungsprogram teilzunehmen?**  ⃝ ja ⃝ nein |

**8)** **Höchster Bildungsabschluss:**

⃝ Obligatorische Schule

⃝ Sekundarstufe II (Berufslehre, FMS, (Berufs-) Matura)

⃝ Tertiärstufe (Eidg. Fachausweis, Höhere Fachschule, Uni)

**10) Migrationshintergrund**

⃝ Kein Migrationshintergrund

⃝ Migrationshintergrund 1. Generation (Pat. im Ausland geboren)

⃝ Migrationshintergrund 2. Generation (Pat. in CH geboren, mind. 1 Elternteil im Ausland geboren)

**Vielen Dank, dass wir Sie zu diesem Thema befragen durften.**

__________________________________________________________________________________

Nachtrag (Verständnisschwierigkeiten, Ergänzungen seitens Patient oder Interviewer):

________________________

________________________

(Verweis, letzte Seite -> Abschluss Bogen, kein zurück)
